# Odorant receptor phylogeny confirms conserved channels for sex pheromone and host plant signals in tortricid moths

**DOI:** 10.1002/ece3.6458

**Published:** 2020-06-30

**Authors:** Francisco Gonzalez, Felipe Borrero‐Echeverry, Júlia K. Jósvai, Maria Strandh, C. Rikard Unelius, Miklós Tóth, Peter Witzgall, Marie Bengtsson, William B. Walker

**Affiliations:** ^1^ Department to Plant Protection Biology Swedish University of Agricultural Sciences Alnarp Sweden; ^2^ ChemTica Internacional Heredia Costa Rica; ^3^ Corporación Colombiana de Investgación Agropecuaria Agrosavia Mosquera Colombia; ^4^ Plant Protection Institute CAR Budapest Hungary; ^5^ Molecular Ecology and Evolution Lab Department of Biology Lund University Lund Sweden; ^6^ Faculty of Health and Life Sciences Linnaeus University Kalmar Sweden; ^7^ Faculty of Forestry and Wood Sciences Czech University of Life Sciences Prague Czech Republic

**Keywords:** behavior‐modifying chemicals, kairomone, Lepidoptera, olfaction, reproductive behavior, semiochemical, sustainable insect control, Tortricidae

## Abstract

The search for mates and food is mediated by volatile chemicals. Insects sense food odorants and sex pheromones through odorant receptors (ORs) and pheromone receptors (PRs), which are expressed in olfactory sensory neurons. Molecular phylogenetics of ORs, informed by behavioral and functional data, generates sound hypotheses for the identification of semiochemicals driving olfactory behavior. Studying orthologous receptors and their ligands across taxa affords insights into the role of chemical communication in reproductive isolation and phylogenetic divergence. The female sex pheromone of green budworm moth *Hedya nubiferana* (Lepidoptera, Totricidae) is a blend of two unsaturated acetates, only a blend of both elicits male attraction. Females produce in addition codlemone, which is the sex pheromone of another tortricid, codling moth *Cydia pomonella*. Codlemone also attracts green budworm moth males. Concomitantly, green budworm and codling moth males are attracted to the host plant volatile pear ester. A congruent behavioral response to the same pheromone and plant volatile in two tortricid species suggests co‐occurrence of dedicated olfactory channels. In codling moth, one PR is tuned to both compounds, the sex pheromone codlemone and the plant volatile pear ester. Our phylogenetic analysis finds that green budworm moth expresses an orthologous PR gene. Shared ancestry, and high levels of amino acid identity and sequence similarity, in codling and green budworm moth PRs offer an explanation for parallel attraction of both species to the same compounds. A conserved olfactory channel for a sex pheromone and a host plant volatile substantiates the alliance of social and habitat signals in insect chemical communication. Field attraction assays confirm that in silico investigations of ORs afford powerful predictions for an efficient identification of behavior‐modifying semiochemicals, for an improved understanding of the mechanisms of host plant attraction in insect herbivores and for the further development of sustainable insect control.

## Graphical abstract

*Hedya nubiferana* (a tortricid moth on apple and other rosacean plants) is attracted to the sex pheromone and to the plant‐derived kairomone of codling moth (another tortricid moth feeding on apple). A conserved odorant receptor is the basis for this coincidence, which demonstrates the interaction of social signals and food cues in insect herbivores.

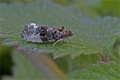

## 1 INTRODUCTION

Olfactory perception of food cues and sex signals is intimately interconnected in insects (Borrero‐Echeverry, Bengtsson, Nakamuta, & Witzgall, 2018; Conchou et al., 2019; Lebreton et al., 2017; Reddy & Guerrero, 2004; Rouyar et al., 2015; Varela, Avilla, Gemeno, & Anton, 2011). Deciphering the chemicals encoding food and mates is basic to understanding insect ecology and evolution. Moreover, the knowledge of such behavior‐modifying chemicals can be applied for detection and environmentally safe control of insects (Evenden & Silk, 2016; Gregg, Del Socorro, & Landolt, 2018; Reddy & Guerrero, 2010; Ridgway, Silverstein, & Inscoe, 1990; Suckling et al., 2014; Witzgall, Kirsch, & Cork, 2010; Witzgall, Stelinski, Gut, & Thomson, 2008).

New tools for insect management are needed in the wake of a changing climate that accelerates insect invasions and outbreaks, aggravating food insecurity (Deutsch et al., 2018). Recent efforts to deregulate the most toxic compounds have left growers with few efficient insecticides (Chandler et al., 2011; Jactel et al., 2019). The overwhelming majority of insect species, however, do not feed on our food crops. Including pollination services, insects are integral to all terrestrial food webs. The overuse of synthetic pesticides affects nontarget and beneficial insects and other arthropods and is a contributing cause of the biodiversity apocalypse. This has been a point of debate since DDT (Carson, 1962), yet despite this, the currently most widely used insecticides, the neonicotinoids show severe side effects (Chmiel, Daisley, Burton, & Reid, 2019; Longing et al., 2020; Seibold et al., 2019; Wagner, 2020; Yamamuro et al., 2019).

The development of pheromones and other semiochemicals as a species‐specific and environmentally safe alternative to conventional insecticides has always been the rationale for chemical ecology research. Air permeation with synthetic pheromones, for disruption of premating sexual communication, is used against key orchard and forest insects (Reddy & Guerrero, 2010; Witzgall, Kirsch, et al., 2010; Evenden & Silk, 2016). Pheromone lures for specific and sensitive detection are available for hundreds of species. Such lures, in combination with traps, insect pathogens or insecticides, may even achieve population control, when the female sex becomes attracted (El‐Sayed, Suckling, Byers, Jang, & Wearing, 2009; Ridgway et al., 1990; Suckling et al., 2014). In stark contrast to pheromones attracting insects for mating, only few semiochemicals have been identified that attract gravid females for oviposition. Designing female or bisexual lures is therefore a current challenge toward a more widespread use of behavior‐modifying chemicals for insect control.

Identification of many hundreds of sex pheromones, across all insect orders (El‐Sayed, 2019), has been facilitated by a mutual coordination of production and response in both sexes. Pheromones are produced in dedicated glands, produce strong antennal responses, and immediately trigger a sequence of distinctive behaviors.

Identification of semiochemicals, or kairomones, that mediate oviposition behavior meets substantial methodological difficulties. Synthetic plant volatile blends that have been found to attract insect herbivores typically build on compounds found across many plant species (Bruce & Pickett, 2011; Lu, Wang, Wang, Luo, & Qiao, 2015; Najar‐Rodriguez, Galizia, Stierle, & Dorn, 2010; Tasin et al., 2010). The attractant power of such ubiquitous plant volatiles is sometimes faint, compared with sex pheromones.

Some plant compounds, that are unique or characteristic for larval food plants, have been found to mediate significant attractancy. One such key host plant compound is ethyl (E,Z)‐2,4‐decadienoate, pear ester, a bisexual attractant for codling moth *Cydia pomonella* (Lepidoptera, Tortricidae) (Light & Knight, 2005; Light et al., 2001). Pear ester is efficient for population monitoring (Knight, Light, & Chebny, 2013; Knight, Mujica, Herrera, & Tasin, 2019) and for behavioral disruption of codling moth larvae and adults, alone or combined with sex pheromone (Knight & Light, 2013; Knight et al., 2012; Light & Beck, 2012; Light & Knight, 2011). The discovery of pear ester demonstrates the potential of kairomones to both improve pheromone‐based techniques and to design stand‐alone applications. That pear ester is released only in trace amounts from green apples (Gonzalez et al., 2020) underlines that the abundance of volatiles in plant headspace does not correlate with their behavioral saliency. Compounds released in large amounts often stem from main biosynthetic pathways shared by many plants and cannot account for specific host plant finding.

The most widely employed tool for studying plant compounds mediating host attraction is gas chromatography coupled to electroantennographic detection (GC‐EAD). GC‐EAD measures the response of the entire antenna to odorants (Arn, Städler, & Rauscher, 1975), and biases compounds occurring in large amounts in headspace collections. An active compound such as pear ester, on the other hand, has been overlooked in GC‐EAD recordings due to its low abundance.

The discovery of the genetic code of insect odorant receptors (ORs) (Clyne et al., 1999) enables a new approach. The ligand binding specificity of ORs determines the spectrum of volatile chemicals transmitted by OSNs from the antenna to olfactory centers in the brain. Sequencing antennal RNA extracts and gene transcript annotation provides OR expression data and a first functional differentiation, between pheromone receptors (PRs) and ordinary ORs, responding to environmental odorants. Subsequent phylogenetic analysis groups orthologous ORs from related species and provides leads on putative ligands, through comparison with an accumulating database of deorphaned insect ORs (Fleischer, Pregitzer, Breer, & Krieger, 2018; Robertson, 2019; Zhang & Löfstedt, 2015). Single ORs are accordingly a tool of choice to interrogate the plant and microbial odorscape for bioactive compounds. A powerful experimental approach is to express ORs singly in defined sensilla of the antenna of *Drosophila melanogaster* (Dobritsa, Van Naters, Warr, Steinbrecht, & Carlson, 2003; Hallem, Ho, & Carlson, 2004), where they can be addressed with single sensillum electrophysiological recordings, coupled to gas chromatography (GC‐SSR).

In codling moth *Cydia pomonella* (Lepidoptera, Tortricidae), CpomOR3 has been deorphaned, following transcriptome analysis (Bengtsson et al., 2012; Walker, Gonzalez, Garczynski, & Witzgall, 2016) and heterologous expression (Bengtsson et al., 2014; Cattaneo et al., 2017; Wan et al., 2019). The main ligand of CpomOR3, which belongs to the PR clade, is the plant volatile pear ester (Bengtsson et al., 2014; Light & Knight, 2005; Light et al., 2001). A recent assembly of the codling moth genome reveals presence of two paralogues of CpomOR3, which, according to functional characterization in *Xenopus* oocytes, respond to a lesser extent also to codling moth sex pheromone, codlemone (Wan et al., 2019). A seemingly conserved response in a closely related species underscores this deeply rooted interconnection of pheromone and plant volatiles: Green budworm moth *Hedya nubiferana* (Lepidoptera, Tortricidae) is attracted to codlemone (Arn, Schwarz, Limacher, & Mani, 1974; El‐Sayed, 2019) and to pear ester (Jósvai, Koczor, & Tóth, 2016; Schmidt et al., 2007).

We have reinvestigated sex pheromone production by green budworm moth *H*. *nubiferana* females and the male response to codling moth *C*. *pomonella* sex pheromone and to pear ester, in laboratory and field bioassays. A comparative phylogenetic analysis of ORs in the antennal transcriptome of green budworm and codling moth aligns with the behavioral evidence and suggests the presence of a conserved olfactory channel dedicated to these compounds, in both species. This demonstrates how functional characterization of ORs in model species such as codling moth (Bengtsson et al., 2014; Gonzalez, Witzgall, & Walker, 2016), followed by in silico studies of antennal transcriptomes in the taxonomically related species will advance the identification of insect kairomones, and the development of insect management.

## 2 MATERIALS AND METHODS

### 2.1 Insects

**Figure 1:**
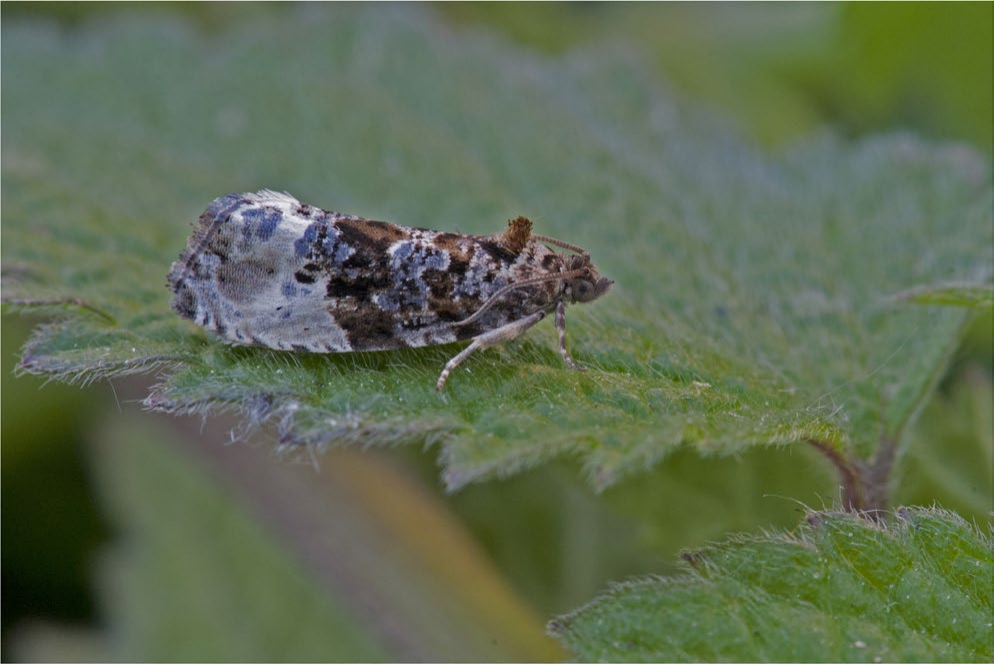
Green budworm moth *Hedya nubiferana* Haworth (*dimidioalba* Retzius) (Lepidoptera, Tortricidae). Photograph by Lubomír Hlásek

Green budworm moth *Hedya nubiferana* Haworth (*dimidioalba* Retzius) (Lepidoptera, Tortricidae) (Figure 1) is a polyphagous leafroller on Rosacean trees and shrubs and co‐occurs with codling moth *Cydia pomonella* on apple, throughout the Northern Hemisphere. The larvae feed on fruit in autumn and on flower buds in the spring (Bradley, Tremewan, & Smith, 1979). For pheromone analysis, last‐instar larvae were field‐collected in apple orchards in Scania (Sweden) during May. Larvae were fed with apple leaves and a semisynthetic agar‐based diet (Rauscher, Arn, & Guerin, 1984). Pupae and adults were kept under a 18:6 hr light–dark cycle in screen cages and were supplied with fresh apple branches and sucrose solution.

### 2.2 Pheromone gland extraction and chemical analysis

Female abdominal sex pheromone glands were dissected at the onset of the calling period, toward the end of the scotophase. Glands of 2‐ to 4‐day‐old females were extracted in batches of 5 to 15 in 7 µl of redistilled hexane for 1 min (Bäckman, Bengtsson, & Witzgall, 1997). Identification of female gland compounds by coupled gas chromatography‐mass spectrometry (GC‐MS) was done on a Hewlett Packard 5970 B instrument, with electron impact ionization (70 eV), interfaced with a Hewlett Packard 5890 GC. Helium was used as carrier gas on a 30 m × 0.25 mm DB‐Wax column (J&W Scientific), programmed from 80°C (hold 2 min) at 10°C/min to 230°C. The compounds were identified by comparing retention times and mass spectra of natural and synthetic compounds. Double bond position was determined by co‐injection with synthetic samples and by evaluation of mass spectra.

### 2.3 Field trapping

The geometric isomers of *E*8,*E*10‐12Ac and *E*8,*E*10‐12OH were synthesized (Witzgall, Bengtsson, Unelius, & Löfqvist, 1993). All other compounds were purchased from S. Voerman (Institute for Pesticide Research, Wageningen, The Netherlands). Purity of synthetic pheromone compounds was ≥96.2% (chemical) and ≥99.7% (isomeric). Compounds in hexanic solution were formulated on red rubber septa (Merck ABS, Dietikon, Switzerland), which were replaced every 2 weeks. Tetra traps (Arn, Rauscher, & Schmid, 1979) were hung in apple trees at eye level and were ca. 5 m apart within one replicate. Traps were placed in untreated apple orchards at Alnarp, Scania (Sweden), and at Halásztelek, Pest county (Hungary), and checked twice a week. Further traps were placed in orchards treated with commercial pheromone dispensers for mating disruption of codling moth. These dispensers were polyethylene tubes containing 87 mg *E*8,*E*10‐12OH, 49 mg 12OH, and 10 mg 14OH (Shin‐Etsu Chemical Co., Tokyo), and they were applied at a rate of 1000/ha. For statistical analysis, trap captures were transformed to log(x + 1) and submitted to a 2‐way ANOVA, followed by Tukey's test.

### 2.4 Wind tunnel

The wind tunnel had a flight section of 63 × 90 × 200 cm (Witzgall et al., 2001). Air was blown by a horizontal fan onto an array of activated charcoal cylinders. The wind tunnel was lit diffusely from above at 6 lux, the wind speed was 30 cm/s, and the temperature ranged from 22 to 24°C. Two‐day‐old males were transferred to glass tubes (2.5 × 12.5 cm) stoppered with gauze before testing. Males were flown individually, in batches of 15. Two batches of 15 males were tested on one day, 1 to 3 hr after onset of the scotophase, each blend was tested four times (*n* = 60 males), on different days. The following types of behavior were recorded: taking flight, flying upwind over 100 cm toward the source, and landing at the source.

### 2.5 Phylogenetic analysis

Sequences of predicted pheromone receptors from C. pomonella (Walker et al., 2016) were used for direct comparison with putative PRs of *H*. *nubiferana* (Gonzalez, Witzgall, & Walker, 2017). All amino acid sequences were aligned using MAFFT online (Katoh, Rozewicki, & Yamada, 2019; version 7.220; http://mafft.cbrc.jp/alignment/server/phylogeny.html) with the FFT‐NS‐i iterative refinement method, with JTT200 scoring matrix, and default parameters. Aligned sequences were used to calculate the best fitting model for comparison in MEGA6 software (Tamura, Stecher, Peterson, Filipski, & Kumar, 2013). The analysis involved 23 amino acid sequences, with a total of 564 positions in the final dataset. An initial tree for the heuristic search was obtained by applying the neighbor‐joining method to a matrix of pairwise distances estimated using a JTT model. Then, a Maximum Likelihood Tree was generated using a JTT matrix‐based model with bootstrap support inferred from 600 replicates. A discrete Gamma distribution was used to model evolutionary rate differences among sites (5 categories (+G, parameter = 3.3624)). The tree is drawn to scale, with branch lengths measured in the number of substitutions per site.

## 3 RESULTS

### 3.1 Sex pheromone identification

Analysis of green budworm moth *H*. *nubiferana* pheromone gland extracts by GC and GC‐MS showed eight further compounds, in addition to the previously identified acetates (Frerot, Priesner, & Gallois, 1979). The major compound codlemone acetate *E*8,*E*10‐12Ac was accompanied by the monounsaturated 8‐ and 10‐dodecenyl acetates, its three geometric isomers (*EZ*, *ZE*, and *Z*8,*Z*10‐12Ac) as well as the analogous alcohol codlemone, *E*8,*E*10‐12OH (Table 1).

**Table 1:** Sex pheromone gland components identified from *H. nubiferana* females by GC/MS

Compound	Short form	ng/female	%
Decyl acetate	10Ac	0.2	2
Dodecyl acetate	12Ac	1.0	15
(*Z*)‐5‐dodecenyl acetate	*Z*5‐12Ac	0.1	2
(*E*)‐8‐dodecenyl acetate	*E*8‐12Ac	0.7	10
(*Z*)‐8‐dodecenyl acetate	*Z*8‐12Ac	3.6	56
(*E*)‐10‐dodecenyl acetate	*E*10‐12Ac	0.7	11
(*Z*)‐10‐dodecenyl acetate	*Z*10‐12Ac	0.1	2
(*Z*,*E*)‐8,10‐dodecadienyl acetate	*Z*8,*E*10‐12Ac	0.3	4
(*E*,*E*)‐8,10‐dodecadienyl acetate	*E*8,*E*10‐12Ac	6.5	100
(*E*,*Z*)‐8,10‐dodecadienyl acetate	*E*8,*Z*10‐12Ac	0.4	6
(*Z*,*Z*)‐8,10‐dodecadienyl acetate	*Z*8,*Z*10‐12Ac	<0.01	trace
(*E*,*E*)‐8,10‐dodecadienol	*E*8,*E*10‐12:OH	0.4	6

Field attraction of *H. nubiferana* males to compounds identified from the female gland confirms that the sex pheromone of *H. nubiferana* is a blend of *E*8,*E*10‐12Ac and *Z*8‐12Ac (Table 2; Frerot et al., 1979). The main compound, *E*8,*E*10‐12Ac, by itself was not attractive, while addition of *Z*8‐12Ac had a strong synergistic effect (*F*(7,72) = 61.95, *p* < .0001). Addition of *E*8‐12Ac further increased male attraction in untreated apple orchards, but the difference was not significant. Blends of *E*8,*E*10‐12Ac and the *∆*10–12 monoenes or the analogous alcohol, codlemone, did not produce significant captures. Adding these compounds to the 3‐component acetate blend slightly diminished trap catch (Table 2).

**Table 2:** Field attraction of *H*. *nubiferana* males to components identified from the female pheromone gland, in untreated apple orchards (*N* = 10) and orchards permeated with codlemone, *E*8,*E*10‐12OH (*N* = 6), June to July 1997

Compound	μg/trap							
*E*8,*E*10‐12:OAc	10	10	10	10	10	10	10	10
*E*8‐12:OAc		1		1	1	1		
*Z*8‐12:OAc			5	5	5	5		
*E*10‐12:OAc					1	1	1	
*Z*10‐12:OAc					0.2	0.2	0.2	
*E*8, *E*10‐12:OH						1		1
	Number of males/trap							
Untreated	0 b	0 b	6.1 a	6.9 a	6.3 a	4.7 a	0.1 b	0 b
Mating disruption	0 a	0 a	0.2 a	0.3 a	0.3 a	0.3 a	0 a	0 a

Note

Means followed by the same letter are not significantly different (Tukey test, *F*(7,72) = 61.95, *p* < .0001).

The gland compounds identified from female glands with no apparent effect on attraction may be biosynthetic by‐products or precursors. A study of the female effluvium will show whether they are released at all, and at which ratio. The full blend of compounds may also carry information that cannot be revealed by a field trapping test.

### 3.2 Attraction to codlemone and pear ester

Wind tunnel observations and a trap test in an apple orchard, adjacent to a pea field, corroborate that codlemone acetate *E*8,*E*10‐12Ac as a single compound does not attract green budworm moth. Attraction of pea moth *C*. *nigricana* confirms that the trap lures released *E*8,*E*10‐12Ac at high isomeric purity (Table 3; Witzgall et al., 1993, 1996). In comparison, traps baited with codlemone alone regularly captured few green budworm moth males, in addition to codling moth. Blends of codlemone and codlemone acetate attract far fewer codling moths and no green budworm moths at all (Table 3).

**Table 3:** Field trapping in apple orchards (*N* = 10) and wind tunnel attraction (*N* = 60) of *H*. *nubiferana* males to compounds identified from the female pheromone gland

Compound	μg/trap				
*E*8,*E*10‐12Ac	10	10	10	1	
*E*8‐12Ac		1	1		
*Z*8‐12Ac		5	5		
*E*8,*E*10‐12OH			10	10	10
	Number of males/trap				
*H. nubiferana*	0 c	57.5 a	53.9 a	0.4 bc	1.4 b
*C. nigricana*	20.1 a	3.4 b	0	0	0
*C. pomonella*	0	0	0	3 b	12.2 a
	Male *H. nubiferana* wind tunnel behavior (%)				
Taking flight	48 a	51 a	47 a	–*	–
Upwind flight	0 b	39 a	33 a	–	–
Landing at source	0 b	22 a	17 a	–	–

Note

Field traps attracted also codling moth *C. pomonella* and pea moth *Cydia nigricana*. Means followed by the same letter are not significantly different (Tukey test, *p* < .05).

* Not tested.

Interestingly, a blend of codlemone and its three geometric isomers significantly increased green budworm moth captures over codlemone alone (Table 4; *F*(7,72) = 2.62, *p* = .04413). In contrast, this isomer blend captured fewer codling moth males (Table 4; *F*(7,72) = 4.22, *p* = .02135; El‐Sayed et al., 1998).

**Table 4:** Field trapping of *H*. *nubiferana* and *C. pomonella* males to the geometric isomers of codlemone *E*8,*E*10‐12OH (*N* = 10)

Compound	μg/trap							
*E*8,*E*10‐12OH	10	10	10	10	10	10	10	10
*E*8,*Z*10‐12OH		0.5	2					2
*Z*8,*E*10‐12OH				0.5	2			2
*Z*8,*Z*10‐12OH						0.5	2	2
*H. nubiferana*	2.0 b	3.5 ab	3.2 ab	2.3 ab	2.1 ab	1.8 b	3.8 ab	6.6 a
*C. pomonella*	8.0 a	9.0 a	4.8 ab	10.5 a	11.9 a	10.2 a	6.4 ab	3.2 b

Note

Means followed by the same letter are not significantly different (Tukey test, *p* < .05).

Green budworm moth has also been reported to respond to pear ester (Jósvai et al., 2016; Schmidt et al., 2007). A further field test in Hungary confirmed this and showed that addition of codlemone to pear ester does not enhance attraction of either sex (Table 5).

**Table 5:** Field trapping of *H*. *nubiferana* males and females with blends of pear ester, ethyl (E,Z)‐2,4‐decadienoate, and codlemone *E*8,*E*10‐12OH (*N* = 4)

Compound	μg/trap			
Pear ester	6.000	6.000	6.000	6.000
*E*8, *E*10‐12OH		1	3	10
	Number of moths/trap			
Males	0.3 a	0.3 a	0.1 a	0.1 a
Females	0.1 a	0.1 a	0.04 a	0.2 a

Note

Means followed by the same letter Means followed by the same letter are not significantly different (Tukey test, *p* < .05).are not significantly different (Tukey test, *p* < .05).

Orchard mating disruption treatments with codlemone strongly diminished attraction of *H. nubiferana* males to synthetic pheromone (Table 2), corroborating a behavioral effect of codlemone via a dedicated olfactory channel. This supports the idea that communication disruption in moths may be achieved with single pheromonal compounds or incomplete pheromone blends (Carde & Minks, 1995; Porcel et al., 2015), which is of practical importance for the implementation of pheromonal control of codling moth and leafrollers in European orchards.

### 3.3 Phylogenetic analysis and antennal expression

*Hedya nubiferana* Haworth and *Hedya dimidioalba* Retzius are synonymous taxonomic names for green budworm moth. The National Center for Biotechnology Information (NCBI) lists OR sequences (including PRs) as "HnubOR##."

**Figure 2:**
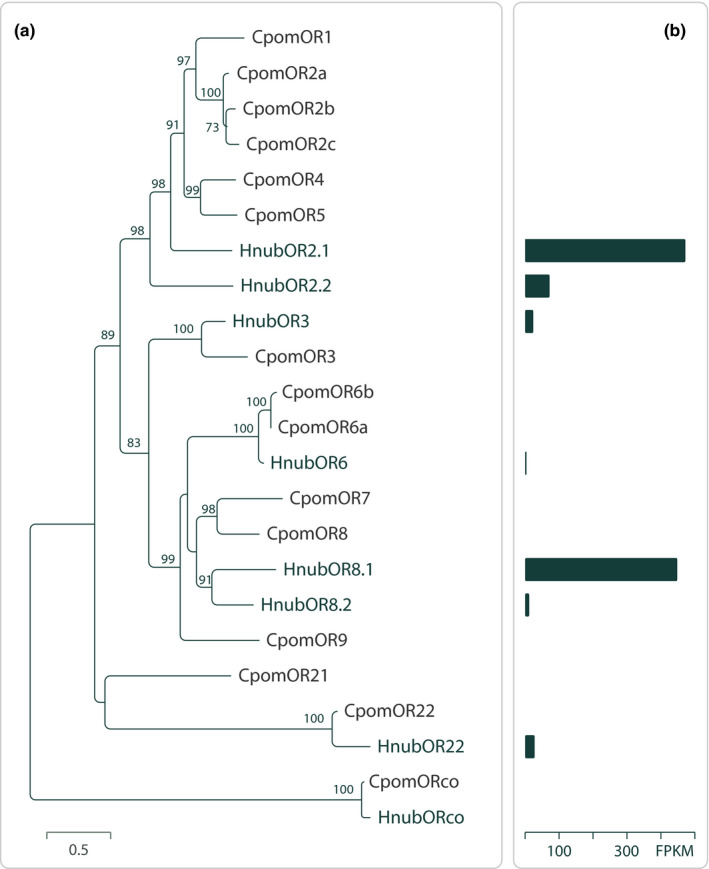
(a) Maximum likelihood unrooted phylogenetic tree of candidate *Hedya nubiferana* (Hnub) pheromone receptors (PR), including PR sequences from codling moth *Cydia pomonella* (Cpom). Node support was assessed with 600 bootstrap replicates, values >70% are shown. (b) *Hedya nubiferana* PR transcript abundance estimates in male antennae. Expression levels quantified by RSEM and indicated as fragments per kilobase of transcript per million reads (FPKM). Data from Walker et al. (2016) and Gonzalez et al. (2017)

**Figure 3:**
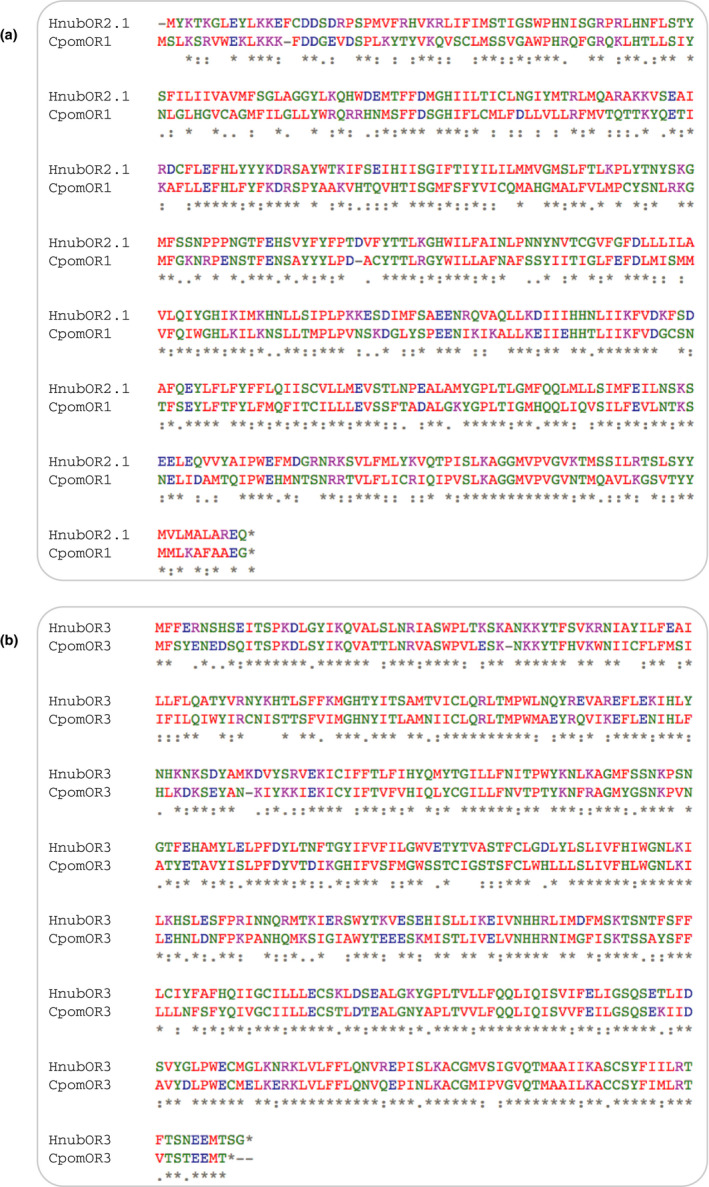
Amino acid alignments of selected *Hedya nubiferana* and *Cydia pomonella* PRs. HnubOR2 and CpomOR1 (a), HnubOR3 and CpomOR3 (b). Sequence data from Walker et al. (2016) and Gonzalez et al. (2017). Amino acid sequence differences are indicated as highly (:) and moderately (.) conservative, and as nonconservative substitutions (blanks). Asterisks indicate identity across both sequences

Predicted putative PRs from *H. nubiferana* displayed orthology to PRs in *Cydia pomonella* (CpomOR3, CpomOR6, and CpomOR22; Figure 2a). Notably, HnubOR6 was >50% similar to CpomOR6. Sequence comparison analysis revealed that CpomOR1 and HnubOR2.1 shared 49% amino acid identity and 66% similarity, while the OR3 orthologs of both species shared 64% and 76% identity and similarity, respectively. Amino acid differences between these putative PRs are observed across the entire length of the protein sequences (Figure 3).

Abundance estimation of the predicted sequences showed that the most highly expressed were HnubOR2.1 and HnubOR8.1 (Gonzalez et al., 2017). The other 3 putative PRs detected in male antennae were one or two orders of magnitude lower (Figure 2b).

## 4 DISCUSSION

### 4.1 In silico identification of semiochemicals for the development of insect control

Semiochemicals are efficient tools for insect control by air permeation and mass trapping (El‐Sayed et al., 2009; Witzgall, Kirsch, et al., 2010), and inform push‐pull techniques and plant breeding (Khan et al., 2014; Stenberg, Heil, Åhman, & Björkman, 2015; Tamiru, Khan, & Bruce, 2015). A current bottleneck and main research challenge is the identification of the chemicals that mediate host plant recognition.

Availability of compounds that attract insects to mating sites, elicit oviposition or feeding in adults and larvae, enables multiple applications. Pear ester, for example, is efficient for monitoring codling moth males and females, is used to supplement pheromone‐based communication disruption, and is a stand‐alone tool for the disruption of larval host‐finding and feeding (Knight & Light, 2013; Knight et al., 2012; Kovanci, 2015; Light, 2016; Light & Beck, 2012; Light & Knight, 2011; Schmidt, Tomasi, Pasqualini, & Ioriatti, 2008). Apple fruit moth, *Argyresthia conjugella*, mates outside apple orchards in forests, which precludes the use of sex pheromone for control. Specific attraction of gravid females to host plant volatiles has been translated into an efficient kairomone lure for monitoring and control (Bengtsson et al., 2006; Knudsen et al., 2008; Knudsen, Norli, & Tasin, 2017; Knudsen & Tasin, 2015).

**Figure 4:**
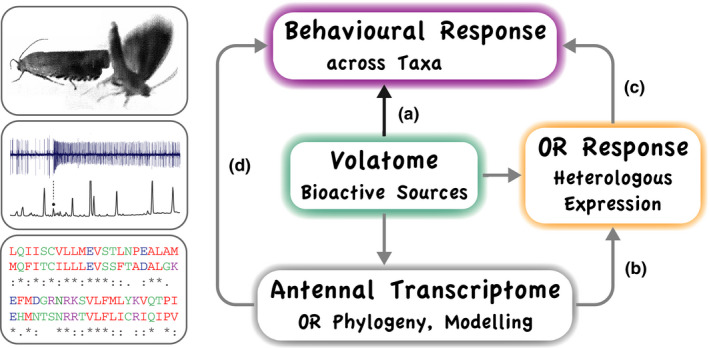
Semiochemical identification workflow, (a) traditional, (b,d) informed by antennal transcriptomes, and (c) by functional characterization of single ORs. (a) Identification of behavior‐modifying chemicals requires analysis of production and response. In insects, screening of candidate compounds in headspace collections, for subsequent behavioral assays, often employs electrophysiological recordings from entire antennae, coupled to a gas chromatograph (GC‐EAD). (b) Transcriptomes reveal olfactory receptors (ORs) expressed in insect antennae, and their phylogenetic relationship with ORs from other species. (c) ORs are expressed singly in cultured cell lines for functional analysis by screening odorant panels. Panels are composed according to chemical analysis of bioactive sources and semiochemical databases. Expression in *Drosophila* olfactory sensory neurons enables GC‐coupled SSR screening of volatile collections (see icon to left). (d) Antennal transcriptomes afford OR sequences, for phylogenetic analysis and topology modeling of ligand binding, and deliver viable hypotheses for accelerated identification of semiochemicals. Behavioral results, across insect taxa, feed back into chemical, transcriptomic, and functional analysis

Chemical analysis of plant or microbial volatomes returns a large number of compounds (Knudsen, Tollsten, & Bergström, 1993; Lemfack et al., 2018; Ljunggren et al., 2019), necessitating careful selection of candidate compounds for subsequent behavioral analysis (Figure 4a). The traditional and most widely used approach is to screen volatile collections, eluting from a gas chromatograph, with the entire insect antenna. GC‐EAD was conceived for sex pheromone identification in moths (Arn et al., 1975), where mutually coordinated production and response lead to distinct male antennal signals to a few bioactive compounds in female pheromone glands.

GC‐EAD suffers, however, from serious bias and produces false positives when screening plant or microbial headspace. Typically, ORs respond, to some extent, to diverse volatiles that are structurally similar to their cognate ligands. Ubiquitous compounds present in large amounts, for example, short aliphatic acetates or alcohols, farnesenes, linalools, and caryophyllenes, invariably elicit an antennal response, generated by the ensemble of olfactory sensory neurons (OSNs) on the antenna, expressing the entire olfactory receptor (OR) repertoire. Their behavioral relevance remains, however, uncertain.

On the other hand, GC‐EAD potentially overlooks active compounds released in small amounts. Pear ester, the strongest known attractant for codling moth, is present in trace amounts in apple headspace and has not been detected by GC‐EAD (Gonzalez et al., 2020). Recordings from single sensilla, instead of the entire antenna, provide higher resolution, but are technically demanding. Reliable replication is a main obstacle for recordings from sensillum types other than *sensilla trichodea*, containing pheromone‐sensitive OSNs. In codling moth, SSR produced conclusive results when investigating sex pheromones, not plant volatiles (Ansebo, Ignell, Löfqvist, & Hansson, 2005; Bäckman et al., 2000).

Antennal transcriptomes and phylogenetic analysis of ORs, followed by heterologous expression (Figure 4b,c), emerge as an opportune methodological advancement to experimentally address single ORs. Functional characterization of ORs expressed in cultured cell lines, such as human embryonic kidney cells or *Xenopus* oocytes, depends, however, on the quality of the odorant panel used, where chemical purity (Schorkopf et al., 2019) and selection of test compounds are main limitations. An experimental difficulty is the aqueous delivery of solubilized volatiles. In comparison, heterologous expression of ORs in select sensory neurons in *Drosophila* enables in vivo single sensillum recordings (SSR). Coupled to a GC, GC‐SSR eliminates differences in volatility of test compounds and makes it even possible to screen entire headspace collections (Dobritsa et al., 2003; Fleischer et al., 2018; Gonzalez et al., 2016; Hallem et al., 2004).

Nonetheless, attempts to deorphan ORs are far from always successful. For example, the orthologous CpomOR19 and SlitOR19 both respond best to indanone analogs (Gonzalez et al., 2015), but their behavioral role remains unclear. An intriguing idea is that the ligand is instead 1,4‐dimethylindanyl acetate (W. Francke, pers. comm.), a rare floral compound (Braunschmid et al., 2017), which is unstable and not available as synthetic standard.

Chemical analysis and functional OR assays deliver candidate compounds for behavioral tests (Figure 4a,c). This selection of compounds may be incomplete, as outlined above, but candidate compounds may anyhow elicit behavioral responses and suffice the criterium of validly identified semiochemicals—while key compounds remain unknown. A prominent example is α‐farnesene, early on identified as a codling moth kairomone (Sutherland & Hutchins, 1972). It is ubiquitously found in most green plants, has some effect on codling moth adult and larvae, but does not encode specific host plant recognition.

In silico identification of OR ligands now emerges as an additional experimental approach and opportune advancement in semiochemical research (Figure 4d). OR expression levels in the sexes, in adult versus larval stages, in combination with phylogenetic analysis and computational approaches (Caballero‐Vidal et al., 2020; Chepurwar, Gupta, Haddad, & Gupta, 2019; De Fouchier et al., 2017), informed by a rapidly accumulating database of deorphaned insect ORs, afford powerful predictions of putative OR ligands and behavior‐modifying chemicals.

Promising targets for future work include, for example, tephritid fruit flies, in view of our thorough knowledge of *Drosophila* ORs (Liu, Smagghe, Lei, & Wang, 2016; Muench & Galizia, 2016) or moths from several families, aided by a rapidly accumulating database of lepidopteran antennal transcriptomes (Cao et al., 2014; Cao, Huang, Shen, Liu, & Wang, 2020; Chang et al., 2017; Corcoran, Jordan, Thrimawithana, Crowhurst, & Newcomb, 2015; Dong, Song, Li, Shi, & Wang, 2016; Du et al., 2018; Feng, Guo, Zheng, Qin, & Du, 2017; Jia et al., 2016; Jia, Zhang, Liu, Wang, & Zhang, 2018; Jiang et al., 2014; Koenig et al., 2015; Li, Du, Li, & Wu, 2015; Park, Withers, Suckling, & Collaboration, 2015; Steinwender, Thrimawithana, Crowhurst, & Newcomb, 2015; Tian et al., 2018; Yang, Cao, Wang, & Liu, 2017; Zeng et al., 2015; Zhang et al., 2013, 2015, 2017; Zhang, Zhang, Wang, & Kong, 2014) Rojas et al. 2018).***

### 4.2 Green budworm moth response to codlemone and pear ester

We here employ a reverse approach, to interpret behavior in the light of transcriptome data and the tortricid OR phylogeny. The empirical finding that green budworm moth *H. nubiferana* males respond to codling moth *C. pomonella* sex pheromone and kairomone, codlemone and pear ester, correlates with the ORs found in antennal transcriptomes.

Functional characterization of CpomOR3, a codling moth OR, has established pear ester as its principal ligand. This was achieved through heterologous expression of CpomOR3 in olfactory sensory neurons of ab3 and T1 antennal sensilla in *Drosophila melanogaster*, followed by single sensillum electrophysiological recordings (SSR) (Bengtsson et al., 2014; Gonzalez et al., 2016), and has meanwhile been corroborated by luminescence assays after expression in human embryonic kidney cells and *Xenopus* oocytes (Cattaneo et al., 2017; Wan et al., 2019). CpomOR3, albeit tuned to a plant volatile compound, is part of the lepidopteran pheromone receptor (PR) clade (Bengtsson et al., 2012, 2014; Walker et al., 2016).

The hypothesis that *H*. *nubiferana* perceives pear ester via HnubOR3 is parsimonious. A PR phylogeny of *H*. *nubiferana* and *C*. *pomonella* (Figure 2a), together with sequence similarity analysis (Figure 3), show that CpomOR3 and HnubOR3 are orthologues, which is in line with the behavioral data (Table 5; Jósvai et al., 2016; Schmidt et al., 2007). This compares to the receptor orthologs CpomOR19 and SlitOR19 (*Spodoptera littoralis*). Following functional characterization of SlitOR19, ligand affinity of CpomOR19 was predicted on the basis of amino acid sequence similarity (Gonzalez et al., 2015).

Oriental fruit moth *Grapholita molesta*, although taxonomically closer to *C*. *pomonella* than to *H*. *nubiferana* (Bradley et al., 1979; Regier et al., 2012), is not known to respond to dienic pheromone compounds or pear ester, which is corroborated by PR phylogeny (Gonzalez et al., 2017; Li et al., 2015). The broad host range of *G. molesta* overlaps only partially with *C*. *pomonella* and *H*. *nubiferana* food plants.

### 4.3 Attraction to sex pheromone and codlemone employs distinct olfactory channels

Attraction of green budworm moth *H. nubiferana* to its multicomponent sex pheromone and to codling moth pheromone employs separate olfactory channels. Codlemone *E*8,*E*10‐12OH does not mimic the *H. nubiferana* main pheromone compound codlemone acetate *E*8,*E*10‐12Ac, since codlemone is active as single compound, while codlemone acetate is not (Tables 2, 3). Tortricid moths differentiate analogous alcohol from acetate pheromone compounds at high resolution (Witzgall et al., 1991, 1993, 1996, 2010), probably since the functional groups strongly affect receptor interactions (Bengtsson, Liljefors, Hansson, Löfstedt, & Copaja, 1990). From analysis of PR phylogeny and expression levels in *H. nubiferana* and *C. pomonella* (Figure 2; Walker et al., 2016), we hypothesize that CpomOR1 and HnubOR2.1 are tuned to codlemone, and CpomOR6 and HnubOR8.1 to codlemone acetate (Cattaneo et al., 2017). In codling moth, codlemone acetate is a pheromone synergist or antagonist, when added to the main pheromone compound codlemone at small and large amounts, respectively (Hathaway et al., 1974; Witzgall et al., 2001).

The presence of two pheromone channels in *H. nubiferana* males is reminiscent of the "hopeful monster" (Baker, 2002; Dietrich, 2003) and "asymmetric tracking" (Phelan, 1992) concepts, suggesting that new communication channels arise through saltational shifts in female pheromone production, which are subsequently tracked by the male sex. Such shifts are facilitated by redundancies in the PR repertoire.

Three related species, *H*. *ochroleucana*, *H. pruniana,* and *H. salicella,* are best attracted to the *Z*,*E* isomers of codlemone and codlemone acetate, and *Z*,*E*‐codlemone is active in codling moth (El‐Sayed et al., 1998; Witzgall, Trematerra, Liblikas, Bengtsson, & Unelius, 2010). A candidate PR for *Z*,*E*‐codlemone is HnubOR2.2 (Figure 2). Regarding HnubOR8.1 and HnubOR8.2, which are close to GmolOR1 and GmolOR11 (Gonzalez et al., 2017; Li et al., 2015), we hypothesize that they respond to the minor acetate pheromone components (*Z*)‐ and (*E*)‐8‐dodecenyl acetate (Tables 2, 3), which are main pheromone compounds of Oriental fruit moth *G. molesta* (Carde, Baker, & Carde, 1979).

### 4.4 Interaction of plant volatiles and pheromones

Food and mate finding, the essential components of insect reproductive behavior, depend on a finite number of ORs encoding relevant odor signals. Peripheral olfactory perception employs 39 ORs in the fruit fly *Drosophila melanogaster* (Grabe, Strutz, Baschwitz, Hansson, & Sachse, 2015; Menuz, Larter, Park, & Carlson, 2014), 58 ORs in codling moth *C*. *pomonella* (Walker et al., 2016), and a similar number of ORs in other tortricids (Corcoran et al., 2015; Steinwender et al., 2015, Rojas et al., 2018). Evolution of host specialization in insects is associated with accelerated OR gene loss, combined with strong selection on the remaining, intact ORs (Arguello et al., 2016; McBride & Arguello, 2007; Robertson, 2019; Sánchez‐Gracia, Vieira, & Rozas, 2009). Receptors that are conserved across taxonomic clades, such as CpomOR3 and HnubOR3 (Figure 2; Gonzalez et al., 2017), likely play adaptive roles.

Green budworm moth attraction to pear ester and codlemone is intriguing, because it provides further evidence for the association of olfactory channels dedicated to social and environmental signals in phytophagous insects. Transcriptome data and phylogenetic context confirm this association. CpomOR3 is tuned to the plant volatile pear ester, while it belongs to the pheromone receptor clade (Figures 2a, 3; Bengtsson et al., 2012, 2014; Walker et al., 2016). That PRs respond to pheromones and plant volatiles has even physiological consequences: OR genes with highest sequence similarity tend to be expressed in OSNs that project to neighboring glomeruli in the antennal lobe, facilitating interactions between the circuits encoding these signals (Couto, Alenius, & Dickson, 2005; Krieger et al., 2009; Ramdya & Benton, 2010). This has indeed been confirmed in codling moth, by intracellular recordings from olfactory projection neurons and functional imaging of the antennal lobe, showing a powerful synergistic interaction between codlemone and pear ester (Trona et al., 2013; Trona, Anfora, Bengtsson, Witzgall, & Ignell, 2010).

HnubOR3 has not been deorphaned, but the recent discovery that CpomOR3 responds to pear ester and to a lesser extent also to codlemone (Wan et al., 2019) provides an explanation for consistent attraction of *H*. *nubiferana* to codlemone (Tables 3, 4; Arn et al., 1974). Codling moth *C. pomonella* and *H. nubiferana* both feed on apple, but belong to different tortricid tribes (Bradley et al., 1979; Regier et al., 2012). Occurrence of conserved olfactory genes contributing to mate finding and host plant attraction lends further support to the concept that host plant recognition and sexual communication are interlinked (Borrero‐Echeverry et al., 2018) and that a combination of natural and sexual selection gives rise to reproductive isolation in insect herbivores (Boughman, 2002; Paterson, 1978; Rosenthal, 2017). A more complete analysis of olfactory genes and their behavioral and ecological functions will contribute to the study of phylogenetic divergence in phytophagous insects. Equally rewarding is the perspective that this research also drives the development of semiochemicals for efficient and sustainable insect control.

## CONFLICT OF INTEREST

None declared.

## AUTHOR CONTRIBUTION

**Francisco Gonzalez:** Formal analysis (equal); Investigation (equal); Validation (equal); Writing‐review & editing (equal). **Felipe Borrero‐Echeverry:** Formal analysis (equal); Investigation (equal); Validation (equal); Writing‐review & editing (equal). **Julia K. Josvai:** Investigation; Writing‐review & editing. **Maria Strandh:** Investigation. **Rikard Unelius:** Investigation; Writing‐review & editing. **Miklos Toth:** Investigation. **Peter Witzgall:** Conceptualization (equal); Data curation (equal); Formal analysis; Funding acquisition (equal); Investigation; Project administration (equal); Supervision (equal); Validation; Visualization (equal); Writing‐original draft (equal). **Marie Bengtsson:** Data curation (equal); Funding acquisition (equal); Investigation; Supervision (equal); Validation; Writing‐review & editing. **William B. Walker:** Conceptualization; Data curation; Formal analysis; Investigation; Supervision (equal); Validation; Visualization; Writing‐review & editing.

## Data Availability

Transcriptome raw reads sequence data are available through the NCBI Sequence Read Archive (Accession Number: SRX1741573). Putative pheromone receptor sequences identified from the *H. nubiferana *transcriptome assembly are available through NCBI, and are included in a Transcriptome Shotgun Assembly project that has been deposited at DDBJ/EMBL/GenBank (accession numbers KY283585.1, KY283590.1, and KY283600.1).

## References

[ece36458-bib-0001] Ansebo, L. , Ignell, R. , Löfqvist, J. , & Hansson, B. S. (2005). Responses to sex pheromone and plant odours by olfactory receptor neurons housed in sensilla auricillica of the codling moth, *Cydia pomonella* (Lepidoptera: Tortricidae). Journal of Insect Physiology, 51, 1066–1074. 10.1016/j.jinsphys.2005.05.003 15964591

[ece36458-bib-0002] Arguello, J. R. , Cardoso‐Moreira, M. , Grenier, J. K. , Gottipati, S. , Clark, A. G. , & Benton, R. (2016). Extensive local adaptation within the chemosensory system following *Drosophila melanogaster's* global expansion. Nature Communications, 7, 11855 10.1038/ncomms11855 PMC4910016 27292132

[ece36458-bib-0003] Arn, H. , Rauscher, S. , & Schmid, A. (1979). Sex attractant formulations and traps for the grape moth *Eupoecilia ambiguella* Hb. Mitteilungen Der Schweizer Entomologischen Gesellschaft, 52, 49–55.

[ece36458-bib-0004] Arn, H. , Schwarz, C. , Limacher, H. , & Mani, E. (1974). Sex attractant inhibitors of the codling moth *Laspeyresia pomonella* L. Experientia, 30, 1142–1144. 10.1007/BF01923655 4435116

[ece36458-bib-0005] Arn, H. , Städler, E. , & Rauscher, S. (1975). The electroantennographic detector ‐ a selective and sensitive tool in the gas chromatographic analysis of insect pheromones. Zeitschrift Für Naturforschung, 30c, 722–725. 10.1515/znc-1975-11-1204

[ece36458-bib-0006] Bäckman, A.‐C. , Anderson, P. , Bengtsson, M. , Löfqvist, J. , Unelius, C. R. , & Witzgall, P. (2000). Antennal response of codling moth males, *Cydia pomonella* (L.) (Lepidoptera: Tortricidae), to the geometric isomers of codlemone and codlemone acetate. Journal of Comparative Physiology A, 186, 513–519. 10.1007/s003590000 10947233

[ece36458-bib-0007] Bäckman, A.‐C. , Bengtsson, M. , & Witzgall, P. (1997). Pheromone release by individual females of codling moth, *Cydia pomonella* L. (Lepidoptera: Tortricidae). Journal of Chemical Ecology, 23, 807–815. 10.1023/B:JOEC.0000006412.16914.09

[ece36458-bib-0008] Baker, T. C. (2002). Mechanism for saltational shifts in pheromone communication systems. Proceedings of the National Academy of Science USA, 99, 13368–13370. 10.1073/pnas.222539799 PMC129676 12374851

[ece36458-bib-0009] Bengtsson, J. M. , Gonzalez, F. , Cattaneo, A. M. , Montagne, N. , Walker, W. B. , Bengtsson, M. , … Witzgall, P. (2014). A predicted sex pheromone receptor of codling moth *Cydia pomonella* detects the plant volatile pear ester. Frontiers in Ecology Evolution, 2, 33 10.3389/fevo.2014.00033

[ece36458-bib-0010] Bengtsson, J. M. , Trona, F. , Montagne, N. , Anfora, G. , Ignell, R. , Witzgall, P. , & Jacquin‐Joly, E. (2012). Putative chemosensory receptors of the codling moth, *Cydia pomonella*, identified by antennal transcriptome analysis. PLoS One, 7(2), e31620 10.1371/journal.pone.0031620 22363688 PMC3282773

[ece36458-bib-0011] Bengtsson, M. , Jaastad, G. , Knudsen, G. , Kobro, S. , Bäckman, A.‐C. , Pettersson, E. , & Witzgall, P. (2006). Plant volatiles mediate attraction to host and non‐host plant in apple fruit moth, *Argyresthia conjugella* . Entomolgia Experimentalis Et Applicata, 118, 77–85. 10.1111/j.1570-7458.2006.00359.x

[ece36458-bib-0012] Bengtsson, M. , Liljefors, T. , Hansson, B. S. , Löfstedt, C. , & Copaja, S. V. (1990). Structure‐activity relationships for chain‐shortened analogs of (*Z*)‐5‐decenyl acetate, a pheromone component of the turnip moth, *Agrotis segetum* . Journal of Chemical Ecology, 16, 667–684. 10.1007/BF01016478 24263583

[ece36458-bib-0013] Borrero‐Echeverry, F. , Bengtsson, M. , Nakamuta, K. , & Witzgall, P. (2018). Plant odour and sex pheromone are integral elements of specific mate recognition in an insect herbivore. Evolution, 72, 2225–2233. 10.1111/evo.13571 30095166 PMC6220987

[ece36458-bib-0014] Boughman, J. W. (2002). How sensory drive can promote speciation. Trends in Ecology and Evolution, 17, 571–577. 10.1016/S0169-5347(02)02595-8

[ece36458-bib-0015] Bradley, J. D. , Tremewan, W. G. , & Smith, A. (1979). British tortricoid moths. Tortricidae: Olethreutinae. London, UK: The Ray Society.

[ece36458-bib-0016] Braunschmid, H. , Mükisch, B. , Rupp, T. , Schäffler, I. , Zito, P. , Birtele, D. , & Dötterl, S. (2017). Interpopulation variation in pollinators and floral scent of the lady’s‐slipper orchid *Cypripedium calceolus* L. Arthropod‐Plant Interactions, 11, 363–379. 10.1007/s11829-017-9512-x

[ece36458-bib-0017] Bruce, T. J. A. , & Pickett, J. A. (2011). Perception of plant volatile blends by herbivorous insects ‐ finding the right mix. Phytochemistry, 72, 1605–1611. 10.1016/j.phytochem.2011.04.011 21596403

[ece36458-bib-0018] Caballero‐Vidal, G. , Bouysset, C. , Grunig, H. , Fiorucci, S. , Montagné, N. , Golebiowski, J. , & Jacquin‐Joly, E. (2020). Machine learning decodes chemical features to identify novel agonists of a moth odorant receptor. Scientific Reports, 10(1), 1–9. 10.1038/s41598-020-58564-9 32015393 PMC6997167

[ece36458-bib-0019] Cao, D. , Liu, Y. , Wei, J. , Liao, X. , Walker, W. B. , Li, J. , & Wang, G. (2014). Identification of candidate olfactory genes in *Chilo suppressalis* by antennal transcriptome analysis. International Journal of Biological Science, 10, 846–860. 10.7150/ijbs.9297 PMC4115196 25076861

[ece36458-bib-0020] Cao, S. , Huang, T. , Shen, J. , Liu, Y. , & Wang, G. (2020). An orphan pheromone receptor affects the mating behavior of *Helicoverpa armigera* . Frontires in Physiology, 11, 413 10.3389/fphys.2020.00413 PMC7204811 32425812

[ece36458-bib-0021] Carde, A. M. , Baker, T. C. , & Carde, R. T. (1979). Identification of a four‐component sex pheromone of the female Oriental fruit moth, *Grapholitha molesta* (Lepidoptera: Tortricidae). Journal of Chemical Ecology, 5, 423–427. 10.1007/BF00987927

[ece36458-bib-0022] Carde, R. T. , & Minks, A. K. (1995). Control of moth pests by mating disruption: Successes and constraints. Annual Review of Entomology, 40, 559–585. 10.1146/annurev.en.40.010195.003015

[ece36458-bib-0023] Carson, R. (1962). Silent Spring. Boston, MA: Houghton Mifflin.

[ece36458-bib-0024] Cattaneo, A. M. , Gonzalez, F. , Bengtsson, J. M. , Corey, E. A. , Jacquin‐Joly, E. , Montagne, N. , … Bobkov, Y. V. (2017). Candidate pheromone receptors from the insect pest *Cydia pomonella* respond to pheromone and kairomone components. Scientific Reports, 7, 41105 10.1038/srep41105 28117454 PMC5259778

[ece36458-bib-0025] Chandler, D. , Bailey, A. S. , Tatchell, G. M. , Davidson, G. , Greaves, J. , & Grant, W. P. (2011). The development, regulation and use of biopesticides for integrated pest management. Philosophical Transactions of the Royal Society of London B, 366, 1987–1998. 10.1098/rstb.2010.0390 PMC3130386 21624919

[ece36458-bib-0026] Chang, X. Q. , Nie, X. P. , Zhang, Z. , Zeng, F. F. , Lv, L. , Zhang, S. , & Wang, M. Q. (2017). De novo analysis of the oriental armyworm *Mythimna separata* antennal transcriptome and expression patterns of odorant‐binding proteins. Comparative Biochemistry and Physiology D, 22, 120–130. 10.1016/j.cbd.2017.03.001 28395238

[ece36458-bib-0027] Chepurwar, S. , Gupta, A. , Haddad, R. , & Gupta, N. (2019). Sequence‐based prediction of olfactory receptor responses. Chemical Senses, 44, 693–703. 10.1093/chemse/bjz078 31665762 PMC6837872

[ece36458-bib-0028] Chmiel, J. A. , Daisley, B. A. , Burton, J. P. , & Reid, G. (2019). Deleterious effects of neonicotinoid pesticides on *Drosophila melanogaster* immune pathways. MBio, 10, e01395‐19 10.1128/mBio.01395-19 31575764 PMC6775452

[ece36458-bib-0029] Clyne, P. J. , Warr, C. G. , Freeman, M. R. , Lessing, D. , Kim, J. , & Carlson, J. R. (1999). A novel family of divergent seven‐transmembrane proteins: Candidate odorant receptors in *Drosophila* . Neuron, 22, 327–338. 10.1016/S0896-6273(00)81093-4 10069338

[ece36458-bib-0030] Conchou, L. , Lucas, P. , Meslin, C. , Proffit, M. , Staudt, M. , & Renou, M. (2019). Insect odorscapes: From plant volatiles to natural olfactory scenes. Frontiers in Physiology, 10, 972 10.3389/fphys.2019.00972 31427985 PMC6688386

[ece36458-bib-0031] Corcoran, J. A. , Jordan, M. D. , Thrimawithana, A. H. , Crowhurst, R. N. , & Newcomb, R. D. (2015). The peripheral olfactory repertoire of the lightbrown apple moth, *Epiphyas p* *ostvittana* . PLoS One, 10, e0128596 10.1371/journal.pone.0128596 26017144 PMC4446339

[ece36458-bib-0032] Couto, A. , Alenius, M. , & Dickson, B. J. (2005). Molecular, anatomical, and functional organization of the Drosophila olfactory system. Current Biology, 15, 1535–1547. 10.1016/j.cub.2005.07.034 16139208

[ece36458-bib-0033] De Fouchier, A. , Walker, W. B. , Montagne, N. , Steiner, C. , Binyameen, M. , Schlyter, F. , … Jacquin‐Joly, E. (2017). Functional evolution of Lepidoptera olfactory receptors revealed by deorphanization of a moth repertoire. Nature Communications, 8, 1–11. 10.1038/ncomms15709 PMC5465368 28580965

[ece36458-bib-0034] Deutsch, C. A. , Tewksbury, J. J. , Tigchelaar, M. , Battisti, D. S. , Merrill, S. C. , Huey, R. B. , & Naylor, R. L. (2018). Increase in crop losses to insect pests in a warming climate. Science, 361, 916–919. 10.1126/science.aat3466 30166490

[ece36458-bib-0035] Dietrich, M. (2003). Richard Goldschmidt: Hopeful monsters and other 'heresies'. Nature Reviews Genetics, 4, 68–74. 10.1038/nrg979 12509755

[ece36458-bib-0036] Dobritsa, A. A. , Van Naters, W. V. D. G. , Warr, C. G. , Steinbrecht, R. A. , & Carlson, J. R. (2003). Integrating the molecular and cellular basis of odor coding in the *Drosophila* antenna. Neuron, 37, 827–841. 10.1016/S0896-6273(03)00094-1 12628173

[ece36458-bib-0037] Dong, J. , Song, Y. , Li, W. , Shi, J. , & Wang, Z. (2016). Identification of putative chemosensory receptor genes from the *Athetis dissimilis* antennal transcriptome. PLoS One, 11, e0147768 10.1371/journal.pone.0147768 26812239 PMC4727905

[ece36458-bib-0038] Du, L. , Zhao, X. , Liang, X. , Gao, X. , Liu, Y. , & Wang, G. (2018). Identification of candidate chemosensory genes in *Mythimna separata* by transcriptomic analysis. BMC Genomics, 19, 518 10.1186/s12864-018-4898-0 29973137 PMC6030794

[ece36458-bib-0039] El‐Sayed, A. M. (2019). The pherobase: database of pheromones and semiochemicals. www.pherobase.com

[ece36458-bib-0040] El‐Sayed, A. M. , Suckling, D. M. , Byers, J. A. , Jang, E. B. , & Wearing, C. H. (2009). Potential of “lure and kill” in long‐term pest management and eradication of invasive species. Journal of Economic Entomology, 102, 815–835. 10.1603/029.102.0301 19610395

[ece36458-bib-0041] El‐Sayed, A. , Unelius, R. C. , Liblikas, I. , Löfqvist, J. , Bengtsson, M. , & Witzgall, P. (1998). Effect of codlemone isomers on codling moth (Lepidoptera: Tortricidae) male attraction. Environmental Entomology, 27, 1250–1254. 10.1093/ee/27.5.1250

[ece36458-bib-0042] Evenden, M. L. , & Silk, P. J. (2016). The influence of Canadian research on semiochemical‐based management of forest insect pests in Canada. The Canadian Entomologist, 148, S170–S209. 10.4039/tce.2015.17

[ece36458-bib-0043] Feng, B. , Guo, Q. , Zheng, K. , Qin, Y. , & Du, Y. (2017). Antennal transcriptome analysis of the piercing moth *Oraesia emarginata* (Lepidoptera: Noctuidae). PLoS One, 12, e0179433 10.1371/journal.pone.0179433 28614384 PMC5470721

[ece36458-bib-0044] Fleischer, J. , Pregitzer, P. , Breer, H. , & Krieger, J. (2018). Access to the odor world: Olfactory receptors and their role for signal transduction in insects. Cellular and Molecular Life Sciences, 75, 485–508. 10.1007/s00018-017-2627-5 28828501 PMC11105692

[ece36458-bib-0045] Frerot, B. , Priesner, E. , & Gallois, M. (1979). A sex attractant for the green budworm moth, *Hedya nubiferana* . Zeitschrift Für Naturforschung, 34c, 1248–1252. 10.1515/znc-1979-1229

[ece36458-bib-0047] Gonzalez, F. , Bengtsson, J. M. , Walker, W. B. , Rodrigues Sousa, M. F. , Cattaneo, A. M. , Montagné, N. , … Bengtsson, M. (2015). A conserved odorant receptor detects the same 1‐indanone analogs in a tortricid and a noctuid moth. Frontires in Ecology and Evolution, 3, 131 10.3389/fevo.2015.00131

[ece36458-bib-0048] Gonzalez, F. , Sousa, M. , Conchou, L. , Walker, W. B. , Chakraborty, A. , Karlsson, M. , … Witzgall, P. (2020). An endophytic yeast odorant mediates codling moth attraction to apple (submitted).

[ece36458-bib-0049] Gonzalez, F. , Witzgall, P. , & Walker, W. B. (2016). Protocol for heterologous expression of insect odourant receptors in *Drosophila* . Frontiers in Ecology Evolution, 4, 24 10.3389/fevo.2016.00024

[ece36458-bib-0050] Gonzalez, F. , Witzgall, P. , & Walker, W. B. (2017). Antennal transcriptomes of three tortricid moths reveal putative conserved chemosensory receptors for social and habitat olfactory cues. Scientific Reports, 7, 41829 10.1038/srep41829 28150741 PMC5288797

[ece36458-bib-0051] Grabe, V. , Strutz, A. , Baschwitz, A. , Hansson, B. S. , & Sachse, S. (2015). Digital in vivo 3D atlas of the antennal lobe of *Drosophila melanogaster* . Journal of Comparative Neurology, 523, 530–544. 10.1002/cne.23697 25327641

[ece36458-bib-0052] Gregg, P. C. , Del Socorro, A. P. , & Landolt, P. J. (2018). Advances in attract‐and‐kill for agricultural pests: Beyond pheromones. Annual Review of Entomology, 63, 453–470. 10.1146/annurev-ento-031616-035040 29058978

[ece36458-bib-0053] Hallem, E. A. , Ho, M. G. , & Carlson, J. R. (2004). The molecular basis of odor coding in the *Drosophila* antenna. Cell, 117, 965–979. 10.1016/j.cell.2004.05.012 15210116

[ece36458-bib-0054] Hathaway, D. O. , McGovern, T. P. , Beroza, M. , Moffitt, H. R. , McDonough, L. M. , & Butt, B. A. (1974). An inhibitor of sexual attraction of male codling moths to a synthetic sex pheromone and virgin females in traps. Environmental Entomology, 3, 522–524. 10.1093/ee/3.3.522

[ece36458-bib-0055] Jactel, H. , Verheggen, F. , Thiéry, D. , Escobar‐Gutiérrez, A. J. , Gachet, E. , & Desneux, N. , Neonicotinoids Working Group (2019). Alternatives to neonicotinoids. Environment International, 129, 423–429. 10.1016/j.envint.2019.04.045 31152983

[ece36458-bib-0056] Jia, X.‐J. , Wang, H.‐X. , Yan, Z.‐G. , Zhang, M.‐Z. , Wei, C.‐H. , Qin, X.‐C. , … Du, Y.‐L. (2016). Antennal transcriptome and differential expression of olfactory genes in the yellow peach moth, *Conogethes punctiferalis* (Lepidoptera: Crambidae). Scientific Reports, 6, 29067 10.1038/srep29067 27364081 PMC4929561

[ece36458-bib-0057] Jia, X. , Zhang, X. , Liu, H. , Wang, R. , & Zhang, T. (2018). Identification of chemosensory genes from the antennal transcriptome of Indian meal moth *Plodia interpunctella* . PLoS One, 13, e0189889 10.1371/journal.pone.0189889 29304134 PMC5755773

[ece36458-bib-0058] Jiang, X. J. , Guo, H. , Di, C. , Yu, S. , Zhu, L. , Huang, L. Q. , & Wang, C. Z. (2014). Sequence similarity and functional comparisons of pheromone receptor orthologs in two closely related *Helicoverpa* species. Insect Biochemistry and Molecular Biology, 48, 63–74. 10.1016/j.ibmb.2014.02.010 24632377

[ece36458-bib-0059] Jósvai, J. K. , Koczor, S. , & Tóth, M. (2016). Traps baited with pear ester and acetic acid attract both sexes of *Hedya nubiferana* (Lepidoptera: Tortricidae). Journal of Applied Entomology, 140, 81–90. 10.1111/jen.12216

[ece36458-bib-0060] Katoh, K. , Rozewicki, J. , & Yamada, K. D. (2019). MAFFT online service: Multiple sequence alignment, interactive sequence choice and visualization. Briefings in Bioinformatics, 20, 1160–1166. 10.1093/bib/bbx108 28968734 PMC6781576

[ece36458-bib-0061] Khan, Z. R. , Midega, C. A. O. , Pittchar, J. O. , Murage, A. W. , Birkett, M. A. , Bruce, T. J. A. , & Pickett, J. A. (2014). Achieving food security for one million sub‐Saharan African poor through push‐pull innovation by 2020. Philosophical Transactions of the Royal Society B, 369, 20120284 10.1098/rstb.2012.0284 PMC3928888 24535391

[ece36458-bib-0062] Knight, A. L. , & Light, D. M. (2013). Adding microencapsulated pear ester to insecticides for control of *Cydia pomonella* (Lepidoptera: Tortricidae) in apple. Pest Management Science, 69, 66–74. 10.1002/ps.3363 22807277

[ece36458-bib-0063] Knight, A. , Light, D. , & Chebny, V. (2013). Monitoring codling moth (Lepidoptera: Tortricidae) in orchards treated with pear ester and sex pheromone combo dispensers. Journal of Applied Entomology, 137, 214–224. 10.1111/j.1439-0418.2012.01715.x

[ece36458-bib-0064] Knight, A. L. , Mujica, V. , Herrera, S. L. , & Tasin, M. (2019). Addition of terpenoids to pear ester plus acetic acid increases catches of codling moth (Lepidoptera: Tortricidae). Journal of Applied Entomology, 143, 942–947. 10.1111/jen.12682

[ece36458-bib-0065] Knight, A. L. , Stelinski, L. L. , Hebert, V. , Gut, L. , Light, D. , & Brunner, J. (2012). Evaluation of novel semiochemical dispensers simultaneously releasing pear ester and sex pheromone for mating disruption of codling moth (Lepidoptera: Tortricidae). Journal of Applied Entomology, 136, 79–86. 10.1111/j.1439-0418.2011.01633.x

[ece36458-bib-0066] Knudsen, G. K. , Bengtsson, M. , Kobro, S. , Jaastad, G. , Hofsvang, T. , & Witzgall, P. (2008). Discrepancy in laboratory and field attraction of apple fruit moth *Argyresthia conjugella* to host plant volatiles. Physiological Entomology, 33, 1–6. 10.1111/j.1365-3032.2007.00592.x

[ece36458-bib-0067] Knudsen, G. K. , Norli, H. R. , & Tasin, M. (2017). The ratio between field attractive and background volatiles encodes host‐plant recognition in a specialist moth. Frontiers in Plant Science, 8, 2206 10.3389/fpls.2017.02206 29312430 PMC5744616

[ece36458-bib-0068] Knudsen, G. K. , & Tasin, M. (2015). Spotting the invaders: A monitoring system based on plant volatiles to forecast apple fruit moth attacks in apple orchards. Basic and Applied Ecology, 16, 354–364. 10.1016/j.baae.2015.03.006

[ece36458-bib-0069] Knudsen, J. T. , Tollsten, L. , & Bergström, L. G. (1993). Floral scents: A checklist of volatile compounds isolated by head‐space techniques. Phytochemistry, 33, 253–280. 10.1016/0031-9422(93)85502-I

[ece36458-bib-0070] Koenig, C. , Hirsh, A. , Bucks, S. , Klinner, C. , Vogel, H. , Shukla, A. , … Grosse‐Wilde, E. (2015). A reference gene set for chemosensory receptor genes of *Manduca sexta* . Insect Biochemistry and Molecular Biology, 66, 51–63. 10.1016/j.ibmb.2015.09.007 26365739

[ece36458-bib-0071] Kovanci, O. B. (2015). Co‐application of microencapsulated pear ester and codlemone for mating disruption of *Cydia pomonella* . Journal of Pest Science, 88, 311–319. 10.1007/s10340-014-0619-x

[ece36458-bib-0072] Krieger, J. , Gondesen, I. , Forstner, M. , Gohl, T. , Dewer, Y. , & Breer, H. (2009). HR11 and HR13 receptor‐expressing neurons are housed together in pheromone‐responsive sensilla trichodea of male *Heliothis virescens* . Chemical Senses, 34, 469–477. 10.1093/chemse/bjp010 19289532

[ece36458-bib-0073] Lebreton, S. , Borrero‐Echeverry, F. , Gonzalez, F. , Solum, M. , Wallin, E. , Hedenström, E. , … Witzgall, P. (2017). A *Drosophila* female pheromone elicits species‐specific long‐range attraction via an olfactory channel with dual specificity for sex and food. BMC Biology, 15, 88 10.1186/s12915-017-0427-x 28962619 PMC5622430

[ece36458-bib-0074] Lemfack, M. C. , Gohlke, B. O. , Toguem, S. M. T. , Preissner, S. , Piechulla, B. , & Preissner, R. (2018). mVOC 2.0: A database of microbial volatiles. Nucleic Acids Research, 46, D1261–D1265. 10.1093/nar/gkx1016 29106611 PMC5753297

[ece36458-bib-0075] Li, G. , Du, J. , Li, Y. , & Wu, J. (2015). Identification of putative olfactory genes from the oriental fruit moth *Grapholita molesta* via an antennal transcriptome analysis. PLoS One, 10(11), e0142193 10.1371/journal.pone.0142193 26540284 PMC4635014

[ece36458-bib-0076] Light, D. M. (2016). Control and monitoring of codling moth (Lepidoptera: Tortricidae) in walnut orchards treated with novel high‐load, low‐density “meso” dispensers of sex pheromone and pear ester. Environmental Entomology, 45, 700–707. 10.1093/ee/nvw017 27018424

[ece36458-bib-0077] Light, D. M. , & Beck, J. J. (2012). Behavior of codling moth (Lepidoptera: Tortricidae) neonate larvae on surfaces treated with microencapsulated pear ester. Environmental Entomology, 41, 603–611. 10.1603/EN11273 22732619

[ece36458-bib-0078] Light, D. M. , & Knight, A. (2005). Specificity of codling moth (Lepidoptera: Tortricidae) for the host plant kairomone, ethyl (2*E*,4*Z*)‐2,4‐decadienoate: Field bioassays with pome fruit volatiles, analogue, and isomeric compounds. Journal of Agricultural and Food Chemistry, 53, 4046–4053. 10.1021/jf040431r 15884837

[ece36458-bib-0079] Light, D. M. , & Knight, A. L. (2011). Microencapsulated pear ester enhances insecticide efficacy in walnuts for codling moth (Lepidoptera: Tortricidae) and navel orangeworm (Lepidoptera: Pyralidae). Journal of Economic Entomology, 104, 1309–1315. 10.1603/EC11058 21882697

[ece36458-bib-0080] Light, D. M. , Knight, A. L. , Henrick, C. A. , Rajapaska, D. , Lingren, B. , Dickens, J. C. , … Campbell, B. C. (2001). A pear‐derived kairomone with pheromonal potency that attracts male and female codling moth, *Cydia pomonella* (L.). Naturwissenschaften, 88, 333–338. (10.1007/s001140100243) 11572014 10.1007/s001140100243

[ece36458-bib-0081] Liu, Z. , Smagghe, G. , Lei, Z. , & Wang, J. J. (2016). Identification of male‐and female‐specific olfaction genes in antennae of the Oriental fruit fly (*Bactrocera dorsalis*). PLoS One, 11, e0147783 (10.1371/journal.pone.0147783) 26845547 10.1371/journal.pone.0147783 PMC4741523

[ece36458-bib-0082] Ljunggren, J. , Borrero‐Echeverry, F. , Chakraborty, A. , Lindblom, T. U. , Hedenström, E. , Karlsson, M. , … Bengtsson, M. (2019). Yeast volatomes differentially effect larval feeding in an insect herbivore. Appl Environm Microbiol, 85, e01761–e1819. 10.1128/AEM.01761-19 PMC6803314 31444202

[ece36458-bib-0083] Longing, S. D. , Peterson, E. M. , Jewett, C. T. , Rendon, B. M. , Discua, S. A. , Wooten, K. J. , … McIntyre, N. E. (2020). Exposure of foraging bees (Hymenoptera) to neonicotinoids in the U.S. southern high plains. Environmental Entomology. 10.1093/ee/nvaa003 32025712

[ece36458-bib-0084] Lu, P. F. , Wang, R. , Wang, C. Z. , Luo, Y. Q. , & Qiao, H. L. (2015). Sexual differences in electrophysiological and behavioral responses of *Cydia molesta* to peach and pear volatiles. Entomologia Experimentalis Et Applicata, 157, 279–290. 10.1111/eea.12362

[ece36458-bib-0085] McBride, C. S. , & Arguello, J. R. (2007). Five *Drosophila* genomes reveal nonneutral evolution and the signature of host specialization in the chemoreceptor superfamily. Genetics, 177, 1395–1416. 10.1534/genetics.107.078683 18039874 PMC2147975

[ece36458-bib-0086] Menuz, K. , Larter, N. K. , Park, J. , & Carlson, J. R. (2014). An RNA‐seq screen of the *Drosophila* antenna identifies a transporter necessary for ammonia detection. PLoS Genetics, 10, e1004810 10.1371/journal.pgen.1004810 25412082 PMC4238959

[ece36458-bib-0087] Muench, D. , & Galizia, C. G. (2016). DoOR 2.0‐Comprehensive mapping of *Drosophila* melanogaster odorant responses. Scientific Reports, 6, 21841 10.1038/srep21841 26912260 PMC4766438

[ece36458-bib-0088] Najar‐Rodriguez, A. J. , Galizia, C. G. , Stierle, J. , & Dorn, S. (2010). Behavioral and neurophysiological responses of an insect to changing ratios of constituents in host plant‐derived volatile mixtures. Journal of Experimental Biology, 213, 3388–3397. 10.1242/jeb.046284 20833933

[ece36458-bib-0089] Park, K. C. , Withers, T. M. , Suckling, D. M. , & Collaboration, B. B. B. (2015). Identification of olfactory receptor neurons in *Uraba lugens* (Lepidoptera: Nolidae) and its implications for host range. Journal of Insect Physiology, 78, 33–46. 10.1016/j.jinsphys.2015.04.010 25937382

[ece36458-bib-0090] Paterson, H. (1978). More evidence against speciation by reinforcement. South African Journal of Science, 74, 369–371.

[ece36458-bib-0091] Phelan, P. L. (1992). Evolution of sex pheromones and the role of assymetric tracking In Roitberg B. D., & Isman M. B. (Eds.), Insect chemical ecology: An evolutionary approach (pp. 265–314). New York, NY: Chapman and Hall.

[ece36458-bib-0092] Porcel, M. , Sjöberg, P. , Swiergiel, W. , Dinwiddie, R. , Rämert, B. , & Tasin, M. (2015). Mating disruption of *Spilonota ocellana* and other apple orchard tortricids using a multispecies reservoir dispenser. Pest Management Science, 71, 562–570. 10.1002/ps.3844 24916099

[ece36458-bib-0093] Ramdya, P. , & Benton, R. (2010). Evolving olfactory systems on the fly. Trends in Genetics, 26, 307–316. 10.1016/j.tig.2010.04.004 20537755

[ece36458-bib-0094] Rauscher, S. , Arn, H. , & Guerin, P. (1984). Effects of dodecyl acetate and Z‐10‐tridecenyl acetate on attraction of *Eupoecilia ambiguella* males to the main sex pheromone component, Z‐9‐Dodecenyl acetate. Journal of Chemical Ecology, 10, 253–264. 10.1007/BF00987853 24318494

[ece36458-bib-0095] Reddy, G. V. , & Guerrero, A. (2004). Interactions of insect pheromones and plant semiochemicals. Trends in Plant Science, 9, 253–261. 10.1016/j.tplants.2004.03.009 15130551

[ece36458-bib-0096] Reddy, G. V. , & Guerrero, A. (2010). New pheromones and insect control strategies. Vitamins and Hormones, 83, 493–519. 10.1016/S0083-6729(10)83020-1 20831959

[ece36458-bib-0097] Regier, J. C. , Brown, J. W. , Mitter, C. , Baixeras, J. , Cho, S. , Cummings, M. P. , & Zwick, A. (2012). A molecular phylogeny for the leafroller moths (Lepidoptera: Tortricidae) and its implications for classification and life history evolution. PLoS One, 7, e35574 10.1371/journal.pone.0035574 22536410 PMC3334928

[ece36458-bib-0098] Ridgway, R. L. , Silverstein, R. M. , & Inscoe, M. N. (1990). Behavior‐modifying chemicals for insect management: Applications of pheromones and other attractants. New York, NY: Marcel Dekker.

[ece36458-bib-0099] Robertson, H. M. (2019). Molecular evolution of the major arthropod chemoreceptor gene families. Annual Review of Entomology, 64, 227–242. 10.1146/annurev-ento-020117-043322 30312552

[ece36458-bib-0100] Rojas, V. , Jimenez, H. , Palma‐Millanao, R. , Gonzalez‐Gonzalez, A. , Machuca, J. , Godoy, R. , … Venthur, H. (2018). Analysis of the grapevine moth Lobesia botrana antennal transcriptome and expression of odorant‐binding and chemosensory proteins. Comparative Biochemistry and Physiology D, 27, 1–12. 10.1016/j.cbd.2018.04.003 29727827

[ece36458-bib-0101] Rosenthal, G. G. (2017). Mate choice: The evolution of sexual decision making from microbes to humans. Princeton, NJ: Princeton University Press.

[ece36458-bib-0102] Rouyar, A. , Deisig, N. , Dupuy, F. , Limousin, D. , Wycke, M. A. , Renou, M. , & Anton, S. (2015). Unexpected plant odor responses in a moth pheromone system. Frontiers in Physiology, 6, 148 10.3389/fphys.2015.00148 26029117 PMC4429231

[ece36458-bib-0103] Sánchez‐Gracia, A. , Vieira, F. , & Rozas, J. (2009). Molecular evolution of the major chemosensory gene families in insects. Heredity, 103, 208–216. 10.1038/hdy.2009.55 19436326

[ece36458-bib-0104] Schmidt, S. , Anfora, G. , Ioriatti, C. , Germinara, G. S. , Rotundo, G. , & De Cristofaro, A. (2007). Biological activity of ethyl (E, Z)‐2,4‐decadienoate on different tortricid species: Electrophysiological responses and field tests. Environmental Entomology, 36, 1025–1031. 10.1603/0046-225X(2007)36[1025:BAOEEO]2.0.CO;2 18284724

[ece36458-bib-0105] Schmidt, S. , Tomasi, C. , Pasqualini, E. , & Ioriatti, C. (2008). The biological efficacy of pear ester on the activity of granulosis virus for codling moth. Journal of Pest Science, 81, 29–34. 10.1007/s10340-007-0181-x

[ece36458-bib-0106] Schorkopf, D. L. P. , Molnar, B. P. , Solum, M. , Larsson, M. C. , Millar, J. G. , Karpati, Z. , & Dekker, T. (2019). False positives from impurities result in incorrect functional characterization of receptors in chemosensory studies. Progress in Neurobiology, 181, 101661 10.1016/j.pneurobio.2019.101661 31310789

[ece36458-bib-0107] Seibold, S. , Gossner, M. M. , Simons, N. K. , Blüthgen, N. , Müller, J. , Ambarli, D. , … Weisser, W. W. (2019). Arthropod decline in grasslands and forests is associated with landscape‐level drivers. Nature, 574, 671–674. 10.1038/s41586-019-1684-3 31666721

[ece36458-bib-0109] Steinwender, B. , Thrimawithana, A. H. , Crowhurst, R. N. , & Newcomb, R. D. (2015). Pheromone receptor evolution in the cryptic leafroller species, *Ctenopseustis obliquana* and *C. herana* . Journal of Molecular Evolution, 80, 42–56. 10.1007/s00239-014-9650-z 25252791

[ece36458-bib-0110] Stenberg, J. A. , Heil, M. , Åhman, I. , & Björkman, C. (2015). Optimizing crops for biocontrol of pests and disease. Trends in Plant Science, 20, 698–712. 10.1016/j.tplants.2015.08.007 26447042

[ece36458-bib-0111] Suckling, D. M. , Stringer, L. D. , Stephens, A. E. , Woods, B. , Williams, D. G. , Baker, G. , & El‐Sayed, A. M. (2014). From integrated pest management to integrated pest eradication: Technologies and future needs. Pest Management Science, 70, 179–189. 10.1002/ps.3670 24155254

[ece36458-bib-0112] Sutherland, O. R. W. , & Hutchins, R. F. N. (1972). α‐Farnesene, a natural attractant for codling moth larvae. Nature, 239, 170.

[ece36458-bib-0113] Tamiru, A. , Khan, Z. R. , & Bruce, T. J. (2015). New directions for improving crop resistance to insects by breeding for egg induced defence. Current Opinion in Insect Science, 9, 51–55. 10.1016/j.cois.2015.02.011 32846708

[ece36458-bib-0114] Tamura, K. , Stecher, G. , Peterson, D. , Filipski, A. , & Kumar, S. (2013). MEGA6: Molecular Evolutionary Genetics Analysis version 6.0. Molecular Biology and Evolution, 30, 2725–2729. 10.1093/molbev/mst197 24132122 PMC3840312

[ece36458-bib-0115] Tasin, M. , Bäckman, A.‐C. , Anfora, G. , Carlin, S. , Ioriatti, C. , & Witzgall, P. (2010). Attraction of female grapevine moth to common and specific olfactory cues from 2 host plants. Chemical Senses, 35, 57–64. 10.1093/chemse/bjp082 19959563

[ece36458-bib-0116] Tian, Z. , Sun, L. , Li, Y. , Quan, L. , Zhang, H. , Yan, W. , … Qiu, G. (2018). Antennal transcriptome analysis of the chemosensory gene families in *Carposina sasakii* (Lepidoptera: Carposinidae). BMC Genomics, 19, 544 10.1186/s12864-018-4900-x 30029592 PMC6053724

[ece36458-bib-0117] Trona, F. , Anfora, G. , Balkenius, A. , Bengtsson, M. , Tasin, M. , Knight, A. , … Ignell, R. (2013). Neural coding merges sex and habitat chemosensory signals in an insect herbivore. Proceedings of the Royal Society B, 280, 20130267 10.1098/rspb.2013.0267 23595270 PMC3652457

[ece36458-bib-0118] Trona, F. , Anfora, G. , Bengtsson, M. , Witzgall, P. , & Ignell, R. (2010). Coding and interaction of sex pheromone and plant volatile signals in the antennal lobe of the codling moth *Cydia pomonella* . Journal of Experimental Biology, 213, 4291–4303. 10.1242/jeb.047365 21113011

[ece36458-bib-0119] Varela, N. , Avilla, J. , Gemeno, C. , & Anton, S. (2011). Ordinary glomeruli in the antennal lobe of male and female tortricid moth *Grapholita molesta* (Busck)(Lepidoptera: Tortricidae) process sex pheromone and host‐plant volatiles. Journal of Experimental Biology, 214, 637–645. 10.1242/jeb.047316 21270313

[ece36458-bib-0120] Wagner, D. L. (2020). Insect declines in the Anthropocene. Annual Review of Entomology, 65, 457–480. 10.1146/annurev-ento-011019-025151 31610138

[ece36458-bib-0121] Walker, W. B. , Gonzalez, F. , Garczynski, S. F. , & Witzgall, P. (2016). The chemosensory receptors of codling moth *Cydia pomonella* ‐ expression in larvae and adults. Scientific Reports, 6, 23518 10.1038/srep23518 27006164 PMC4804390

[ece36458-bib-0122] Wan, F. , Yin, C. , Tang, R. , Chen, M. , Wu, Q. , Huang, C. , … Wang, G. (2019). A chromosome‐level genome assembly of *Cydia pomonella* provides insights into chemical ecology and insecticide resistance. Nature Communications, 10, 1–14. 10.1038/s41467-019-12175-9 PMC6748993 31530873

[ece36458-bib-0138] Witzgall, P. , Bengtsson, M. , Buser, H. R. , Chambon, P. J. , Priesner, E. , Wildbolz, T. , & Arn, H. (1991). Sex pheromones of Spilonota ocellana and Spilonota laricana. Entomologia Experimentalis et Applicata, 60, 219–223. 10.1111/j.1570-7458.1991.tb01541.x

[ece36458-bib-0123] Witzgall, P. , Bengtsson, M. , Rauscher, S. , Liblikas, I. , Bäckman, A.‐C. , Coracini, M. , … Löfqvist, J. (2001). Identification of further sex pheromone synergists in the codling moth, *Cydia pomonella* . Entomologia Experimentalis Et Applicata, 101, 131–141. 10.1046/j.1570-7458.2001.00898.x

[ece36458-bib-0124] Witzgall, P. , Bengtsson, M. , Unelius, C. R. , & Löfqvist, J. (1993). Attraction of pea moth Cydia nigricana F. (Lepidoptera: Tortricidae) to female sex pheromone (E, E)‐8,10‐dodecadien‐1‐yl acetate, is inhibited by geometric isomers (E, Z), (Z, E) and (Z, Z). Journal of Chemical Ecology, 19, 1917–1928. 10.1007/BF00983796 24249368

[ece36458-bib-0125] Witzgall, P. , Chambon, J.‐P. , Bengtsson, M. , Unelius, C. R. , Appelgren, M. , Makranczy, G. , … Löfqvist, J. (1996). Sex pheromones and attractants in the Eucosmini and Grapholitini (Lepidoptera, Tortricidae). Chemoecology, 7, 13–23. 10.1007/BF01240633

[ece36458-bib-0126] Witzgall, P. , Kirsch, P. , & Cork, A. (2010). Sex pheromones and their impact on pest management. Journal of Chemical Ecology, 36, 80–100. 10.1007/s10886-009-9737-y 20108027

[ece36458-bib-0127] Witzgall, P. , Stelinski, L. , Gut, L. , & Thomson, D. (2008). Codling moth management and chemical ecology. Annual Review of Entomology, 53, 503–522. 10.1146/annurev.ento.53.103106.093323 17877451

[ece36458-bib-0128] Witzgall, P. , Trematerra, P. , Liblikas, I. , Bengtsson, M. , & Unelius, C. R. (2010). Pheromone communication channels in tortricid moths: Lower specificity of alcohol vs. acetate geometric isomer blends. Bulletin of Entomological Research, 100, 225–230. 10.1017/S0007485309990186 19586577

[ece36458-bib-0129] Yamamuro, M. , Komuro, T. , Kamiya, H. , Kato, T. , Hasegawa, H. , & Kameda, Y. (2019). Neonicotinoids disrupt aquatic food webs and decrease fishery yields. Science, 366, 620–623. 10.1126/science.aax3442 31672894

[ece36458-bib-0130] Yang, S. , Cao, D. , Wang, G. , & Liu, Y. (2017). Identification of genes involved in chemoreception in *Plutella xyllostella* by antennal transcriptome analysis. Scientific Reports, 7, 1–16. 10.1038/s41598-017-11646-7 28931846 PMC5607341

[ece36458-bib-0131] Zeng, F.‐F. , Zhao, Z.‐F. , Yan, M.‐J. , Zhou, W. , Zhang, Z. , Zhang, A. , … Wang, M.‐Q. (2015). Identification and comparative expression profiles of chemoreception genes revealed from major chemoreception organs of the rice leaf folder, *Cnaphalocrocis medinalis* (Lepidoptera: Pyralidae). PLoS One, 10, e0144267 10.1371/journal.pone.0144267 26657286 PMC4676629

[ece36458-bib-0132] Zhang, D. D. , & Löfstedt, C. (2015). Moth pheromone receptors: Gene sequences, function, and evolution. Frontiers in Ecology and Evolution, 3, 105 10.3389/fevo.2015.00105

[ece36458-bib-0133] Zhang, J. , Wang, B. , Dong, S. , Cao, D. , Dong, J. , Walker, W. B. , … Wang, G. (2015). Antennal transcriptome analysis and comparison of chemosensory gene families in two closely related noctuidae moths, *Helicoverpa armigera* and *H*. *assulta* . PLoS One, 10, e0117054 10.1371/journal.pone.0117054 25659090 PMC4319919

[ece36458-bib-0134] Zhang, S.‐F. , Liu, H.‐H. , Kong, X.‐B. , Wang, H.‐B. , Liu, F. , & Zhang, Z. (2017). Identification and expression profiling of chemosensory genes in *Dendrolimus punctatus* Walker. Frontiers in Physiology, 8, 471 10.3389/fphys.2017.00471 28736530 PMC5500615

[ece36458-bib-0135] Zhang, S. , Zhang, Z. , Wang, H. , & Kong, X. (2014). Antennal transcriptome analysis and comparison of olfactory genes in two sympatric defoliators, *Dendrolimus houi* and *Dendrolimus kikuchii* (Lepidoptera: Lasiocampidae). Insect Biochemistry and Molecular Biology, 52, 69–81. 10.1016/j.ibmb.2014.06.006 24998398

[ece36458-bib-0136] Zhang, Y.‐N. , Jin, J.‐Y. , Jin, R. , Xia, Y.‐H. , Zhou, J.‐J. , Deng, J.‐Y. , & Dong, S.‐L. (2013). Differential expression patterns in chemosensory and non‐chemosensory tissues of putative chemosensory genes identified by transcriptome analysis of insect pest the purple stem borer *Sesamia inferens* (Walker). PLoS One, 8, e69715 10.1371/journal.pone.0069715 23894529 PMC3722147

